# Intratumoral Heterogeneity of MAGE-C1/CT7 and MAGE-C2/CT10 Expression in Mucosal Melanoma

**DOI:** 10.1155/2015/432479

**Published:** 2015-06-16

**Authors:** A. Curioni-Fontecedro, R. Pitocco, N. L. Schoenewolf, D. Holzmann, D. Soldini, R. Dummer, S. Calvieri, H. Moch, D. Mihic-Probst, A. Fitsche

**Affiliations:** ^1^Department of Oncology, University Hospital Zurich, 8091 Zurich, Switzerland; ^2^Department of Dermatology, Policlinico Umberto I, University of Rome La Sapienza, 00185 Rome, Italy; ^3^Department of Dermatology, University Hospital Zurich, 8091 Zurich, Switzerland; ^4^Department of Otorhinolaryngology, University Hospital Zurich, 8091 Zurich, Switzerland; ^5^Institute of Surgical Pathology, University Hospital Zurich, 8091 Zurich, Switzerland

## Abstract

Mucosal melanoma is a rare disease, which differs from its cutaneous counterpart genetically and for its clinical behaviour. Moreover this is a heterogeneous disease based on the tissue of origin. As CT7 and CT10 are highly expressed in cutaneous melanoma and are immunogenic in this disease, we analysed their expression throughout the different subtypes of mucosal melanoma and tumor development. We detected a frequent expression of CT7 in primaries and corresponding metastases (55%) as well as for CT10 (30%). This expression resulted to be heterogeneous in the same tumor specimen and moreover influenced by the tissue of origin. Our results support the role of these antigens in immunotherapy for mucosal melanoma.

## 1. Introduction

Cancer-testis (CT) antigen (Ags) represent a family of proteins widely studied in the field of cancer immunotherapy because of their restrictive expression pattern and immunogenicity in cancer patients [1]. In normal tissues, the expression of CT antigen is restricted to germ line tissues (namely, placenta, ovaries, and testis), which express small amounts of HLA molecules, making CT antigen no more recognisable from the immune system. For this reason, CTAgs represent ideal candidates for vaccination strategies in cancer. Although expressed in several cancers, their role in tumorigenesis is however unclear. Of interest is that the aberrant expression of germ line genes in cancer reflects the activation of a program which is silenced in somatic cells; moreover, it is very well known that the gain of genes is one of the driving forces of tumorigenesis [2]. This has been also postulated for different CT antigen, expressed diffusely in malignant tissues and frequently coexpressed as the consequence of a common promoter demethylation [3, 4]. Among the CT antigen, the MAGE family is one of the most extensively investigated antigen so far, with documented expression in several cancers [2]. Between these, MAGE-C1/CT7 (from now on CT7) and MAGE-C2/CT10 (from now on CT10) are highly expressed in cutaneous melanoma (CM), represent strong prognostic markers [5], and spontaneously induce a specific cellular immune response in melanoma patients [6, 7].

Different from the cutaneous counterpart are mucosal melanomas (MM). These are unrelated to ultraviolet light exposure, develop at later age and more frequently at advanced stage, are characterised by a high risk of local recurrence, and develop more often distant metastases. Genetically they differ from the cutaneous counterpart for the rare presence of* Braf* mutations. Of interest is also that MM are a heterogeneous disease and, based on the tissue of origin, genetic differences have been detected as for* c-kit* mutations, which were found in almost half of genital melanoma and nonsinonasal melanoma [8–10].

Due to clinical and genetic differences between CM and MM and heterogeneity of MM, we aimed at evaluating the expression of CT7 and CT10 in MM and their presence throughout the different subtypes and tumor development.

## 2. Materials and Methods

### 2.1. Patients' Population

54 melanoma samples from 33 patients were analysed. From these, 33 out of 54 were primary melanomas of which 12 derived from a gynaecological localization (vulva and vagina), 1 from the anus, 3 from the conjunctiva, and 17 from the sinonasal region (of these, 3 cases were melanoma in situ). From 21/33 patients matching samples were available as follows: primary and metachronous recurrence in 14 cases (8 local and 6 distant) and 7 cases with primary and synchronous lymphonodal metastases. Description of samples and matching recurrence or metastases is reported in Table S1 (see Table S1 in the Supplementary Material available online at http://dx.doi.org/10.1155/2015/432479). Tumor specimens were retrieved from the archives of the Institute for Surgical Pathology Zurich (University Hospital Zurich) and the Department of Dermatology of the University Umberto I, Rome, Italy, between 1996 and 2012. All cases were reviewed from an experienced pathologist (DM). Approval for the use of melanoma tissue was obtained from the official ethical authorities of the Canton Zurich (StV 16-2007, Amendment, 2014). All patients provided a written informed consent in accordance with the Declaration of Helsinki.

### 2.2. Immunohistochemistry

Tissue sections of 2.0 *μ*m were cut, mounted on glass slides, deparaffinised, rehydrated, and stained with hematoxylin-eosin using standard histological techniques. Heavily pigmented melanomas are difficult for immunohistochemical interpretation; therefore, in order to avoid false positive results the slides were bleached before using immunohistochemistry as previously described [11].

For immunohistochemical staining, the Ventana Benchmark automated staining system and Ventana reagents were used (Ventana Medical Systems, Tucson, AZ). Immunohistochemistry was performed as recently described [12, 13]. Primary antibodies against CT7 (clone CT7-33, Dako Cytomation, Dilution 1 : 80, Glostrup, Denmark) and CT10 clone LX-CT10.5, Dilution 1 : 100 [14] were used. Immunohistochemical analysis for CT7 and CT10 was evaluated based on the percentage of positive cells and defined as positive if at least 5% of tumor cells were positive for each of the two antigen [15, 16].

### 2.3. *c-Kit* Mutation Analysis

From 28 melanomas deriving from 19 patients* c-KIT* mutational analysis was known. These data are available from our previous study [10].

### 2.4. Statistical Analysis

Correlations between primary melanomas and their metastases for CT10 or CT7 expression were analyzed using Spearman's rank correlation. CT7 and CT10 expression were compared between different localisation groups using the Mann-Whitney *U* test. *P* values below 0.05 were considered as significant. IBM SPSS Statistics 20 (SPSS Inc., Chicago, IL) was used for statistical analyses. GraphPad Prism 5 was used for Boxplots and Graphs.

## 3. Results

### 3.1. MAGE-C1/CT7 and MAGE-C2/CT10 Expression in Mucosal Melanoma

**Figure 1:**
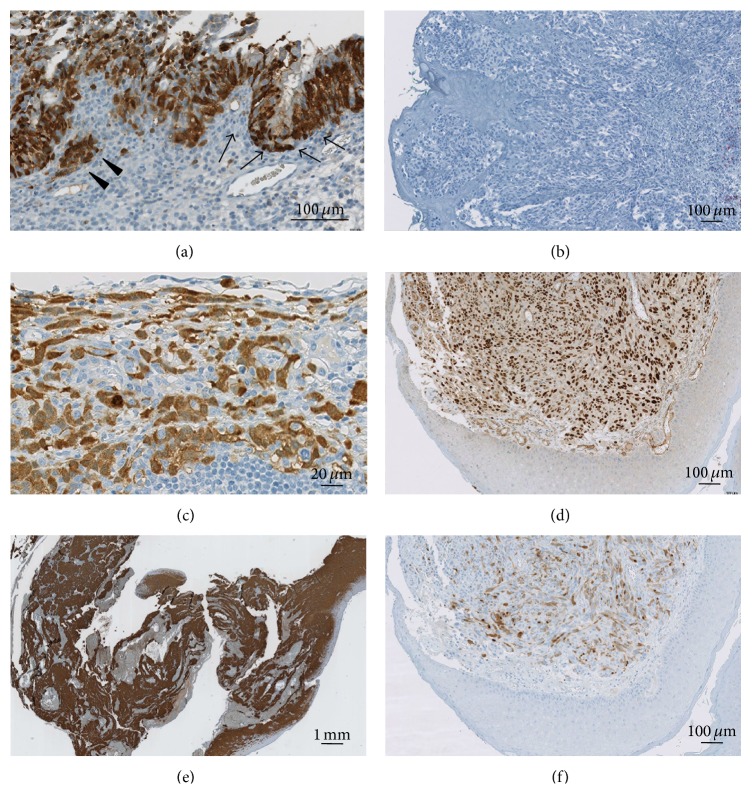
CT7 and CT10 expression in melanoma: primary sinonasal melanoma with positivity for CT7 in the in situ (arrow) and invasive part (arrow head (a)). Maintained CT7 expression in the corresponding metastases (c) and local recurrence (e). Negativity for CT7 in a vaginal melanoma (b). Nuclear positivity for CT10 (d) and cytoplasmic positivity for CT7 (f) on the same anal melanoma.

As previously described [5], we found CT7 expression in the nucleus, cytoplasm, or both compartments in both primary melanoma and metastases in 30 out of 54 (55%) melanoma lesions (Figure 1(a)). There was a significant difference of CT7 expression between sinonasal and gynecological melanoma (*P* = 0.002). Twenty-five out of 32 (78%) sinonasal melanomas stained positive for CT7 in contrast to only 3 out of 17 (18%) of gynecological melanoma samples (Figure 1(b)). CT7 expression was also detected in 1 out of 2 anal melanoma lesions and 1 out of 3 melanomas of the conjunctiva (Table S1(a)).

CT10 expression was detected in 16 of 54 melanomas (30%); of these 13 showed a nuclear staining and 3 a combined nuclear and cytoplasmic expression. In contrast to CT7, we found no significant difference in CT10 expression between sinonasal (30%) and gynecological (26%) melanoma (for details, see Table S1(b)). CT10 expression was also detected in 2 out of 2 anal melanoma lesions and none from the conjunctiva (Table S1(b)).

Coexpression of CT7 and CT10 was detected in 6 out of 33 (18%) primaries and 5 out of 21 (24%) metastases (Figures 1(d) and 1(f)).

### 3.2. Correlation of MAGE-C1/CT7 and MAGE-C2/CT10 Expression in Primary Melanoma and Its Recurrence

Analysis of primary tumors and corresponding recurrence (21 cases) showed a significant correlation of CT7 expression in primary MM and the recurrence (*P* = 0.001; Spearman's correlation coefficient 0,7) as well as of CT10 (*P* = 0.01; Spearman's correlation coefficient 0,6).

**Figure 2:**
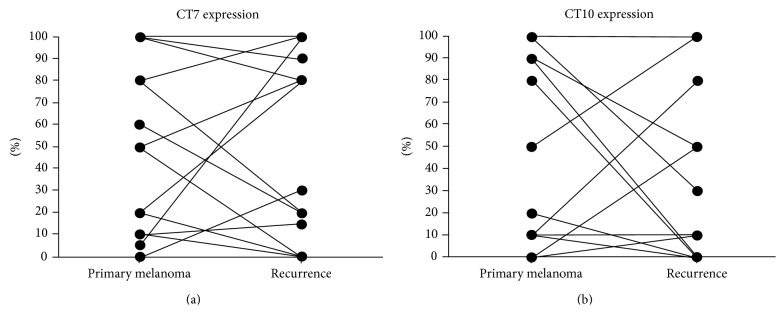
Percentage of CT7 (a) and CT10 (b) melanoma cells in the primary melanoma and corresponding recurrence.

Analysing the matched samples of sinonasal melanomas in detail, only one patient showed CT7 expression in the primary tumor and negative recurrence. In all other patients, if CT7 expression was present in the primary tumor, this was also detected in the recurrence; moreover in five cases an increase of CT7 expression was detected in the recurrence (Figures 1(a), 1(c), and 1(e)). For CT10, 2 patients showed an increased expression in the recurrence, 2 showed a stable expression, and 3 cases, positive in the primary lesion, had negative recurrences (Figures 2(a) and 2(b)).

## 4. Discussion

Mucosal melanoma is a rare disease with aggressive features and, due to occult localization, the diagnosis most frequently occurs in the late stages. This disease differs from the cutaneous counterparts for its biology and genetic features as is the case of BRAF, which is often mutated in cutaneous melanoma and rarely in MM [8, 17]. Moreover, MM does not represent a unique tumor entity but differs due to alternative ontogenesis [8, 18] as, for example, mutations of* KIT* are more often detected in gynecological MM rather than sinonasal differing also clinically in distinct forms, a unilocular and multilocular subtype [19]. Moreover, from a clinical perspective, a major event in MM compared to CM is also the more frequent local recurrences due to the anatomical challenges of a radical resection. For these reasons we focused our interest in MM in order to evaluate the immunogenicity of this rare disease. Indeed, immunotherapy plays a major role in melanoma [20] with extraordinary responses in patients treated with immune checkpoint inhibitors and we have previously demonstrated the spontaneous immunogenicity of cutaneous melanoma by the frequent expression on CT7 and CT10 and spontaneous immune responses to CT7 in these patients. In this study, we analysed the expression of CT7 and CT10 in 54 MM samples and detected a frequent expression of CT7 in primaries and corresponding metastases (55%) as well as of CT10 (30%). This expression resulted to be heterogeneous in the same tumor specimen. This result is very intriguing, because although heterogeneity of melanoma is very well known, MAGE antigen are commonly considered to be coexpressed upon activation of a common promoter [21], especially for CT7 and CT10, which are located next to each other on the X chromosome and share more than 50% of sequence-homology.

This result suggests a possible modulation mechanism by which tumors can escape the immune recognition by switch-on and -off of protein that might also be involved in tumorigenesis and formation of metastases [12, 22–25]. Of importance is that CT7 expression, compared to other vaccine-targets for melanoma [26], does not get lost during tumor progression, stressing the potential role of this antigen for therapeutic purposes and monitoring of immune responses at all stages of disease. To this, we have previously demonstrated and characterized a CT7-specific cellular immune response in melanoma patients [7]. One of these patients was affected by sinonasal melanoma and his tumor sample is included in the current cohort of cases, demonstrating the immunogenicity of CT7 also in mucosal melanoma.

Of interest is that CT7 might represent an ideal candidate for vaccination especially in sinonasal melanomas, as its expression, compared to the gynecological ones, occurs in about 78% of samples compared to the gynecological ones, where its expression is detected in 18% of lesions. This is of major interest as our study demonstrates that CT7 expression is influenced by the tissue of origin and might be modulated by events related to ontogenesis.

In order to get new insights on the mechanism of expression of these genes we intended to define the correlation between CT7 and CT10 expression and the presence of activating KIT mutations; this is based on the frequent activation of the* KIT* gene in MM and the finding that an activated* KIT* may allow MAGE gene expression in mast cell leukemia [27]. In contrast to mast cell leukemia we found no clear-cut correlation between* KIT* mutation and MAGE gene expression: mutational analysis for* KIT* was found in 5/28 available samples and CT7 expression was detected in none and CT10 in 1 of 5 (20%) of these cases (primaries and metastasis). However, due to the very limited number of* KIT* activation positive cases, the relation between* KIT* activation and CT7 and CT10 expression, respectively, in mucosal melanoma is not yet clear and has to be analysed in a larger cohort of patients.

Taken together our findings demonstrate CT7 and CT10 as good therapeutic targets for vaccination strategies and monitoring of immune responses for patients with mucosal, especially sinonasal, melanoma. However, as failure to cancer vaccines due to tumor-associated immunosuppression might occur, possible combinatorial treatments with immune-checkpoint inhibitors would be ideal for future clinical studies.

## Supplementary Material

Supplementary Table 1a. Expression of CT7 in primary and metastases of mucosal melanoma from different anatomical regions. Positivity in more (∗)or less (∗∗)than 20% of tumor cells.
Supplementary Table 1b. Expression of CT10 in primary and metastases of mucosal melanoma from different anatomical regions- Positivity in more (∗)or less (∗∗)than 20% of tumor cells.
