# Erratum to “Intratumoral Heterogeneity of MAGE-C1/CT7 and MAGE-C2/CT10 Expression in Mucosal Melanoma”

**DOI:** 10.1155/2019/5256364

**Published:** 2019-07-22

**Authors:** A. Curioni-Fontecedro, R. Pitocco, N. L. Schoenewolf, D. Holzmann, D. Soldini, R. Dummer, S. Calvieri, H. Moch, A. Fitsche, D. Mihic-Probst

**Affiliations:** ^1^Department of Oncology, University Hospital Zurich, 8091 Zurich, Switzerland; ^2^Department of Dermatology, Policlinico Umberto I, University of Rome La Sapienza, 00185 Rome, Italy; ^3^Department of Dermatology, University Hospital Zurich, 8091 Zurich, Switzerland; ^4^Department of Otorhinolaryngology, University Hospital Zurich, 8091 Zurich, Switzerland; ^5^Institute of Surgical Pathology, University Hospital Zurich, 8091 Zurich, Switzerland

In the article titled “Intratumoral Heterogeneity of MAGE-C1/CT7 and MAGE-C2/CT10 Expression in Mucosal Melanoma” [1], there was an error in the authors' order, as Dr. D. Mihic-Probst should be listed as the last author. The corrected authors' list and affiliations' list are shown above.
