# Supplementary material for: Intratumoral Heterogeneity of MAGE-C1/CT7 and MAGE-C2/CT10 Expression in Mucosal Melanoma
Source: Biomed Res Int. 2015 Jun 16;2015:432479. doi: 10.1155/2015/432479 (PMC4486606; doi:10.1155/2015/432479)
Supplement: Supplementary file 1 — Supplementary Table 1a. Expression of CT7 in primary and metastases of mucosal melanoma from different anatomical regions. Positivity in more (∗)or less (∗∗)than 20% of tumor cells. Supplementary Table 1b. Expression of CT10 in primary and metastases of mucosal melanoma from different anatomical regions- Positivity in more (∗)or less (∗∗)than 20% of tumor cells. [file 432479.f1.docx]

**Supplementary Tables**

| **CT7 expression** | **Localisation** | | | |
| --- | --- | --- | --- | --- |
|  | Gynecological  (*/**) | Sinonasal  (*/**) | Anal  (*/**) | Ocular  (*/**) |
| Primary | 2/12 | 13/17 | 1/1 | 1/3 |
| Metastases | 1/5 | 12/15 | 0/1 |  |
| **Total** | 3/17 | 25/32 | ½ | 1/3 |

Table 1a

Expression of CT7 in primary and metastases of mucosal melanoma from different

anatomical regions. Positivity in more (*)or less (**)than 20% of tumor cells

| **CT10 expression** | **Localisation** | | | |
| --- | --- | --- | --- | --- |
|  | Gynecological  (*/**) | Sinonasal  (*/**) | Anal  (*/**) | Ocular  (*/**) |
| Primary | 2/12 | 6/17 | 1/1 | 0/3 |
| Metastases | 2/5 | 4/15 | 1/1 |  |
| **Total** | 4/17 | 10/32 | 2/2 | 0/3 |

Table 1b

Expression of CT10 in primary and metastases of mucosal melanoma from different

anatomical regions- Positivity in more (*)or less (**)than 20% of tumor cells
